# Glucose Metabolism in Burns—What Happens?

**DOI:** 10.3390/ijms22105159

**Published:** 2021-05-13

**Authors:** Silviu Constantin Badoiu, Daniela Miricescu, Iulia-Ioana Stanescu-Spinu, Alexandra Ripszky Totan, Silvia Elena Badoiu, Michel Costagliola, Maria Greabu

**Affiliations:** 1 Department of Anatomy and Embryology, Faculty of Medicine, Carol Davila University of Medicine and Pharmacy, 8 Eroii Sanitari Blvd, 050474 Bucharest, Romania; silviu.badoiu@umfcd.ro; 2 Department of Plastic and Reconstructive Surgery, Life Memorial Hospital, 365 Grivitei Street, 010719 Bucharest, Romania; 3 Department of Biochemistry, Faculty of Dental Medicine, Carol Davila University of Medicine and Pharmacy, 8 Eroii Sanitari Blvd, 050474 Bucharest, Romania; alexandra.totan@umfcd.ro (A.R.T.); maria.greabu@umfcd.ro (M.G.); 4 Faculty of Medicine, Carol Davila University of Medicine and Pharmacy, 8 Eroii Sanitari Blvd, 050474 Bucharest, Romania; silvia.badoiu@stud.umfcd.ro; 5 Department of Plastic and Reconstructive Surgery, Faculté de Médicine Toulouse-Rangueil 3, Université Toulouse III—Paul Sabatier, Rue de Languedoc, CEDEX 04, 31000 Toulouse, France; costagliola.m@wanadoo.fr

**Keywords:** glucose metabolism, burns, hypermetabolic state, hyperglycemia, insulin resistance

## Abstract

Severe burns represent an important challenge for patients and medical teams. They lead to profound metabolic alterations, trigger a systemic inflammatory response, crush the immune defense, impair the function of the heart, lungs, kidneys, liver, etc. The metabolism is shifted towards a hypermetabolic state, and this situation might persist for years after the burn, having deleterious consequences for the patient’s health. Severely burned patients lack energy substrates and react in order to produce and maintain augmented levels of glucose, which is the fuel “ready to use” by cells. In this paper, we discuss biological substances that induce a hyperglycemic response, concur to insulin resistance, and determine cell disturbance after a severe burn. We also focus on the most effective agents that provide pharmacological modulations of the changes in glucose metabolism.

## 1. Introduction

Burns represent one of the most severe forms of trauma and also are a major public health problem [1,2]. Annually, about 300 million patients are affected by burns, the fourth most frequent kind of trauma after traffic accidents, violent incidents, and falls [3]. Severe burns involve more than 30–40% of the total body surface area (TBSA) and affect the entire human body (all its systems), inducing long hospitalization and increasing mortality [4]. In the past decade, several studies demonstrated that burns affecting only 10% of the TBSA might induce alterations similar to those developed after burns involving more than 30% of the TBSA [5].

Severely burned patients react with a systemic inflammatory response and a hypermetabolic response. The systemic inflammatory response is totally disproportionate and abnormal [6]. It begins in the first hours after the burn trauma [6,7] and persists for about one month and a half [8]. In severe burns, the inflammatory response is triggered by the initial trauma, but subsequently, it might be reinitiated several times by surgical debridement of the burn wound, by septic complications, by sleep deprivation, or by exposure to a cold environment [9]. In such situations, when the inflammatory response cannot be mitigated, it induces multiple organ failure and even death [10]. From the biochemical point of view, the systemic inflammatory pathway intersects with the trajectory leading to a hypermetabolic state, [11] having some common hallmarks: elevated levels of acute-phase proteins, cytokines, and chemokines [5], such as tumor necrosis factor-α (TNFα), interleukin 1β (IL-1β), interleukin 6 (IL-6), insulin-like growth factor 1 (IGF-1) [12], increased serum levels of catecholamines, etc. Unlike the inflammatory response which usually persists for only 5–6 weeks after the initial insult, the hypermetabolic state in severe burns can persist for up to 36 months [12].

The amplitude of the inflammatory and metabolic responses of patients depends on the burn depth and percentage of the TBSA affected by the burn [5], etiology of the burn and presence of an inhalation injury [6], presence of other traumatic injuries, preexistent health conditions of the patient, comorbidities, previous medication, age of the patient, time elapsed from the moment of the acute event till presentation to the hospital etc. [9].

The hypermetabolic state is generated by severe energy deprivation at the cellular level. In order to satisfy the huge energetic need, at a certain moment after the burn, the metabolism shifts towards increased glycolysis, glycogenolysis, gluconeogenesis, lipolysis, proteolysis [5].

Among the most prominent metabolic alterations are persistent hyperglycemia and insulin resistance [12,13] which greatly contribute to increased morbidity and mortality of severe burns [4,14].

## 2. Glucose Metabolism in Burns

Glucose metabolism regulation is quite strict in normal conditions [15]. After food intake, glucose serum levels increase. Circulating glucose is transported into cells, where glucose may be used as a source of energy (glycolysis, oxidative phosphorylation) or stored (glycogenesis, lipogenesis). In specific situations, the excess of glucose may be used in thermogenesis [15]. When glucose serum levels decrease (fasting, starvation), glycogenolysis accompanies the switch of metabolism towards using fats and eventually proteins for glucose synthesis (gluconeogenesis) and energy production [16,17,18].

After severe burns, patient’s metabolic status and glucose metabolism go through significant changes. In burns involving more than 20% of the TBSA, the metabolic response has been described as having two phases: the “ebb” phase and the “flow” phase [19,20,21]. 

In the “ebb” phase, which lasts the first 2–4 days post-burn, the metabolic rate is reduced, the circulating blood volume, cardiac output, and tissue perfusion are decreased, and the oxygen consumption drops [9,19,20,21]. It partially superposes on the so-called burn shock phase, which is typical for severe burns and looks initially like any hypovolemic shock:

- decreased plasma volume due to extravasation into the burn wound and later into normal tissues because of vasodilation, increased blood flow, increased vascular permeability (produced by histamine, prostaglandin E2, prostacyclin, thromboxane A2, thromboxane B2, bradykinin, serotonin, reactive oxygen species (ROS)) [22,23,24];
- decreased cardiac output determined by the reduced preload and the circulating myocardial depressant factor [22,23,24];
- decreased renal filtration rate with decreased urine output (caused by reduced perfusion of the kidneys) [22,23,24];
- increased systemic vascular resistance which accentuates the tendency to reduce tissue perfusion [22,23,24].

The key metabolic change in this phase is represented by reduced metabolic rate or “hypometabolism” [19]. It is believed that severe mitochondrial dysfunction and accentuated endoplasmic reticulum stress generate an important decrease of all the metabolic processes at the cell level [20].

Some of the factors that produce hemodynamic and inflammatory reactions (catecholamines, IL-1, ROS, etc.) initiate metabolic changes, too, in the “ebb” phase. These factors (and others) continue to act and progressively amplify the subsequent metabolic changes in the “flow” phase [4].

The “flow” phase is initiated, amplified and maintained by the continuing action of stress hormones (cortisol, catecholamines, glucagon) and cytokines (TNFα, IL-1β, IL-6) [9].

Therefore, towards the fifth day, the patient reaches an inflammatory hyperdynamic and hypermetabolic state called the “flow” phase [18,25].

This phase is characterized by:

- tachycardia and increased blood pressure due to the action of catecholamines and stimulation of the sympathetic nervous system [25];
- peripheral insulin resistance [18];
- increased glycolysis [5];
- augmented glycogenolysis [5];
- accentuated gluconeogenesis [5];
- elevated lipolysis [5];
- persistent proteolysis [5].

Most authors consider these acute modifications an “adaptive response” [18] that aims at covering the severely increased energy requirements and maintaining the body temperature which contributes to survival in the short term.

The consequences are:

- increased thermogenesis [13];
- increased resting energy expenditure (REE) [13];
- muscle wasting with decreased lean body mass [26].

However, if the hypermetabolic response and its consequences persist, as is the case with severe burns, patients become exhausted and lose their ability to respond [9]. They develop multiple organ dysfunction which may lead to death [10].

The alterations in metabolic status/glucose metabolism impact the wound healing process, too. Briefly, insulin resistance, increased protein catabolism, and persistent hyperglycemia determine:

- immunodeficiency and increased risk of systemic and/or burn wound infections;
- delayed wound healing;
- poor quality scars;
- complicated graft taking.

All these contribute to increased hospital stay, augmented mortality, and the need for close follow-up of the survivors of major burns after hospital discharge [27]. The specific alterations in the “ebb” phase and in the “flow” phase are summarized for a view at a glance in Table 1:

### 2.1. The Key Role of Proinflammatory Cytokines in Hypermetabolic Response

Several proinflammatory cytokines, such as tumor necrosis factor-α (TNFα), interleukin 1β (IL-1β), and interleukin 6 (IL-6), play a key role in the pathogenesis of the systemic inflammatory response and the hypermetabolic state of severely burned patients by being involved in alterations of all kinds of metabolism, including glucose metabolism.

#### 2.1.1. Tumor Necrosis Factorα

Tumor necrosis factor-α (TNFα), a well-known inflammatory cytokine, is produced by macrophages/monocytes during the systemic inflammatory response which accompanies a severe burn [28,29]. The levels of this biomarker of inflammation that binds to specific receptors which activate the NF-κB-dependent signaling pathway are increased only in the acute phase post-burn [18,30]. Moreover, the levels of TNFα and of its receptors correlate with the severity of the burn [29].

Apart from its role in inflammation and apoptosis, TNFα stimulates formation of ROS (Reactive Oxygen Species) and increases the rate of lipolysis in burns [31,32]. These two last actions have consequences upon glucose metabolism, too. By stimulating the formation of ROS, TNFα might indirectly interfere with glycolysis and oxidative phosphorylation because ROS from the mitochondria can cause dysregulation in glycolysis and vice versa [33].

Moreover, by increasing lipolysis, TNFα increases the release of free fatty acids (FFAs) [34] which influence glucose metabolism and induce insulin resistance. TNFα is not only an acute-phase inflammatory cytokine, but also an adipose tissue-secreted cytokine [35] produced by adipocytes and cells of the vascular stroma of the adipose tissue [35] and acts upon the transcription process in the fat tissue and in the liver [36]. In the adipose tissue, TNFα inhibits expression of the genes that have a role in the uptake and storage of circulating glucose and FFAs, having an inhibitor consequence upon lipogenesis [36]. Furthermore, it stimulates lipolysis by activating the mitogen-activated protein kinase module (MAP kinase module) [34]. TNFα activates two of the three protein kinases that form the MAP kinase signaling module: extracellular signal-related kinase (ERK) and the mitogen-activated protein kinase (MAPK) [34]. These kinases enter the nucleus and influence transcription of specific genes, including those controlling the cell glucose uptake [30].

**Figure 1:**
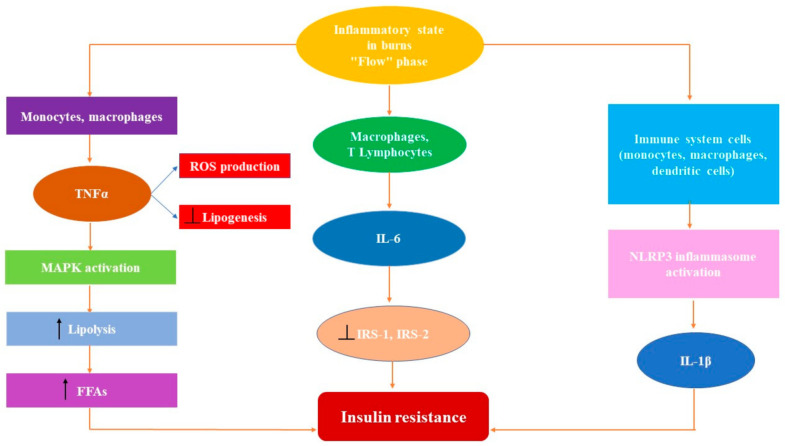
The inflammatory state in the "flow" phase of severe burns. Monocytes and macrophages secrete the tumor necrosis factor-α (TNFα), leading to overproduction of reactive oxygen species (ROS) and inhibition of lipogenesis. Moreover, increased TNFα levels lead to mitogen activation of the protein kinase (MAPK), resulting in increased lipolysis and the release of a higher amount of free fatty saturated acids (FFAs). IL-6 is released by T lymphocytes and macrophages, blocking insulin receptor substrates 1 and 2 (IRS-1, IRS-2). Immune system cells stimulate the release of IL-1β by the NLRP3 inflammasome. The augmented release of all three proinflammatory cytokines contributes to insulin resistance. “
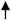
” shows an increase; “
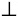
” indicates an inhibitory effect.

In the liver, TNFα blocks expression of the genes involved in glucose uptake and in the oxidation of fatty acids. On the other hand, it amplifies expression of the genes that control lipogenesis, stimulating the synthesis of fatty acids and cholesterol [36]. In addition, it has been proven that TNFα induces insulin resistance by interfering with insulin signaling directly and indirectly [37]. Directly, TNFα prevents Tyr (Tyrosine) phosphorylation of IRS-1 (insulin receptor substrate-1) and promotes Ser (Serine) phosphorylation of IRS-1. These actions blunt the transmission of the insulin signal towards intracellular signaling pathways [37,38]. Indirectly, by increasing the lipolysis, TNFα increases the intracellular fatty acids and the circulating levels of FFAs which stimulate the Ser phosphorylation of IRS-1, the result being restrained insulin signaling [39]. Therefore, in burns, aside from being a proinflammatory cytokine, TNFα contributes to insulin resistance and increases the level of blood glucose and of FFAs, which are indicators of a hypermetabolic state (Figure 1).

#### 2.1.2. Interleukin 1β (IL-1β)

Interleukin 1β is an important proinflammatory cytokine which is produced within immune system cells (especially in monocytes, macrophages, and dendritic cells), though not only there, in response to infections and trauma [40]. Various pathogens carry associated molecular patterns (PAMPs) which stimulate the production of pro-IL-1β by activating pattern recognition receptors (PRRs) [40,41]. Pro-IL-1β is activated by caspase-1 which is secreted in an inactive form (pro-caspase-1) and autoactivates itself after its recruitment to high-molecular-weight complexes called inflammasomes [42].

Inflammasomes are activated by pathogens, PAMPs, or DAMPs (disease-associated molecular patterns) or by “environmental irritants.” The best characterized inflammasome until now is called the NLRP3 inflammasome due to its node-like receptor domain [42]. It appears that this inflammasome is activated in burns by ROS (which result from mitochondrial disfunction) and by increased levels of saturated fatty acids [43].

Apart from its role in inflammation, IL-1β appears to be connected to hyperglycemia by acting upon the spinal cord, upon the sympathetic nervous system, and upon hypothalamus [44,45,46]. IL-1β levels are elevated in chronic pain and induce hyperglycemia by stimulating the glucocorticoid system [44] and the stress axis [40,41]. Such effects were demonstrated in experiments on mice, but not in humans [44,45,46].

#### 2.1.3. Interleukin 6 (IL-6)

The IL-6 level increases early in burn patients, being produced by activated macrophages and T lymphocytes, and induces both proinflammatory and anti-inflammatory effects [47]. Persistently higher levels of IL-6 are associated with an increased risk of infection and death [11,48]. Administration of IL-6 to healthy subjects induces a metabolic response similar to the "hypercatabolic state” described in severely burned patients: elevated serum concentrations of glucose and FFAs and increased resting energy expenditure [49]. It has been hypothesized that the hypermetabolic effect of IL-6 is a consequence of its action on the liver, with the induction of an acute phase response [18]. The liver produces acute-phase proteins such as C-reactive proteins, serum amyloid A, haptoglobin, fibrinogen, protease inhibitors, transport proteins, etc. [50].

In healthy human subjects, IL-6 has an increasing effect upon glucose metabolism in skeletal muscles by amplifying glucose transport in muscle cells, glycogenogenesis, and glucose oxidation [51]. It has been proven that the elevated uptake of glucose in human skeletal muscle cells by IL-6 is not a consequence of insulin-stimulated glucose transport [51]. Moreover, the insulin signaling pathway might not be influenced at all by IL-6 in human skeletal muscles in basal conditions [51].

In the liver, as well as in skeletal muscles, IL-6 activates multiple signaling pathways, such as the MAPK pathway, the PI3K (phosphoinositide 3-kinase) pathway, and the STAT1 and STAT3 (signal transducer and activator of transcription) pathways [50]. Activation of the PI3K pathway determines activation of the AKT (serine/threonine protein kinase) that phosphorylates GSK3 (glycogen synthase kinase 3) and inactivates it. Consequently, there is an increase in the cellular uptake of glucose and glycogen synthesis, which lead to a decrease of blood sugar levels [52]. STAT3 determines the upregulation of SOCS3 (suppressor of cytokine signaling 3), which inhibits the phosphorylation of insulin substrate receptors 1 and 2, thus preventing the insulin-mediated activation of PKB (protein kinase B) or AKT [50]. Therefore, STAT pathway activation acts as a negative feedback loop upon PKB (AKT) activation by IL-6 [53].

In severe burns, IL-6 contributes to insulin resistance in the liver and skeletal muscles, acting upon insulin receptor substrates [11]. The increased resting energy expenditure in these patients might be partially explained by the direct action of IL-6 upon the central nervous system [54]. This interleukin exerts its action in the brain through trans-signaling [55], the capacity of IL-6 to act upon the cells that do not express the membrane-bound IL-6 receptor [56].

### 2.2. Stress Crosstalk from Burns to Glucose Metabolism

#### 2.2.1. Catecholamines

In severe burns, catecholamine levels increase in the circulating blood early after the trauma and induce a hyperdynamic circulatory state and a hypercatabolic state [13] characterized by elevated blood pressure, resting energy expenditure and glycogenolysis, and decreased glycogenesis [13,57,58]. They influence the lipid and protein metabolism, too [13]. Moreover, high levels of circulating epinephrine and norepinephrine persist for up to 18–24 months after the acute event and contribute to the so-called chronic shock, characterized, among other things, by long periods of insulin resistance and hyperglycemia [12,59].

Epinephrine has major metabolic effects, especially on the adipose tissue, liver, and muscles, which, together with the respiratory, cardiovascular, renal, ocular, digestive tract, skeletal muscle, adipose tissue effects, appear after the catecholamines bind to adrenergic receptors [60,61]. These are tissue-specific and cell-specific effects [62]. The adrenergic receptors (α_1,2_ and β_1,2,3_) are members of the class of G protein-coupled receptors (which contains more than 800 members) [63]. G protein-coupled receptors (GPCR) are characterized by a generic structure: each receptor is a transmembrane single-chain polypeptide with a characteristic spatial conformation that looks like a cylinder with an extracellular domain (where the ligand binds) and an intracellular domain (that binds to or is already coupled with a G protein). The catecholamine molecule (for instance, adrenaline) is a ligand for the adrenergic receptors and, after ligation, it determines a change in the spatial conformation of the aforementioned receptor and activates the G protein [63].

G proteins are regulatory proteins—they are heterotrimeric GTP-binding proteins (guanosine triphosphate-binding proteins) [64]. G proteins are characterized by GPRC and plasma membrane proteins specificity [63]. Each G protein is composed of three subunits: α, β, and γ. The α subunit is bound to GDP (guanosine diphosphate) in the unstimulated (basal) state; the GDP-αβγ heterotrimer loses GDP in the activated state; GTP binds the α subunit that changes its conformation; GTP-α subunit dissociates from the βγ subunit; each of them acts on various substrates, having specific effects. Consequently, cAMP (cyclic adenosine monophosphate) (a secondary messenger) or Ca^2+^ (a common intracellular mediator) concentrations change in the cytosol [62].

**Figure 2:**
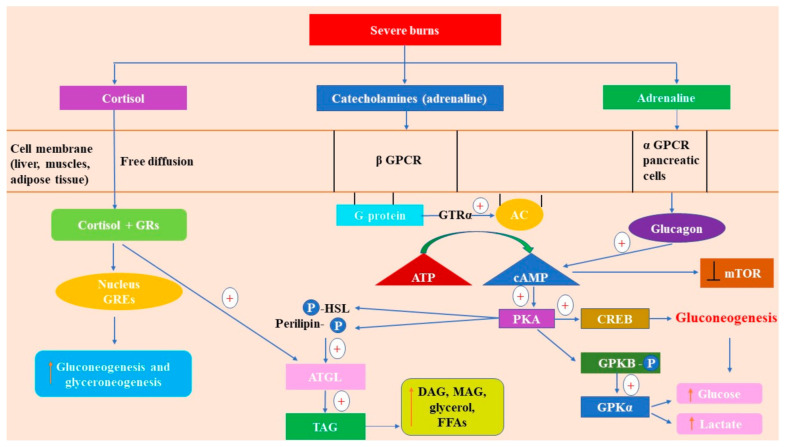
Stress hormones in severe burns. The released catecholamines bind to the β receptor (a G protein-coupled receptor (GPCR)), leading to adenyl cyclase (AC) activation; further, the second messenger, cyclic adenosine monophosphate (cAMP), is formed by adenosine triphosphate hydrolysis (ATP), leading to protein kinase B (PKB) activation. PKA phosphorylates glycogen phosphorylase b kinase (GPKb) to its active form, GPKa, conducing to an increased level of glucose and lactate. Moreover, PKA stimulates phosphorylation of the CREB (cAMP response element-binding protein), promoting gluconeogenesis. PKA indirectly activates the adipocyte triglyceride lipase (ATGL) after the phosphorylation of perilipin and hormone-sensitive lipase (HSL), with the release of diacylglycerol (DAG), monoacylglycerol (MAG), glycerol, and FFAs. In α-pancreatic cells, adrenaline stimulates the release of glucagon which activates cAMP and inhibits the mammalian target of rapamycin (mTOR). Cortisol binds to glucocorticoid-responsive elements (GREs) that enter the nucleus and promote gene transcription of the key enzymes from gluconeogenesis and glyceroneogenesis. “
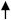
” marks an increase; “
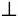
” shows an inhibitory effect; “
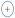
” indicates stimulatory effects.

Typically, the GTP-α subunit stimulates the adenylyl cyclase (AC), resulting increased synthesis of cAMP from ATP. This synthesized cAMP activates protein kinase A (PKA), which increases the phosphorylation of glycogen phosphorylase b kinase (GPKb) to its active a form (GPKa) (Figure 2). GPK is activated by glucagon, too, which is another hormone with increased levels in burns [65]. GPK mobilizes the glycogen in the liver and muscles (glycogen breakdown) and increases the glycolysis, leading to hyperglycemia. The glycogen phosphorylase b kinases in the liver and muscles are isoenzymes [66,67].

Most metabolic effects of catecholamines are due to the activation of β-adrenergic receptors. In severe burns, the effect of catecholamines inhibiting oxidative phosphorylation and promoting the anaerobic glycolysis in muscles is enhanced. Finally, catecholamines increase circulating levels of glucose and lactate [62].

Indirect metabolic effects result from the action of catecholamines upon the pancreas: stimulation of β_2_ receptors enhances the production of insulin, while stimulation of α_2_ receptors reduces the production of insulin and the global effect is inhibition of insulin secretion [68]. By stimulating the β adrenergic receptors of the α-pancreatic cells, catecholamines increase glucagon secretion [69]. Another indirect influence of catecholamines on glucose metabolism in severe burns (which represent a major stress) is the stimulation of β receptors in the adipose tissue. The activity of PKA increases through the signaling pathway involving adenylyl cyclase activation followed by increased synthesis of cAMP [62], consequently phosphorylating the regulatory protein perilipin and the hormone-sensitive lipase (HSL) [70,71]. Furthermore, PKA indirectly activates the adipocyte triglyceride lipase (ATGL) [72,73]. After PKA phosphorylates perilipin-1 in adipocytes, CGI-58 (a coactivator also known as α/β-hydrolase domain-containing protein 5, ABHD5) is liberated from perilipin-1 and activates the ATGL [74]. The consequence is increased lipolysis: triacylglycerol (TAG) is hydrolyzed to diacylglycerol (DAG), monoacylglycerol (MAG), glycerol, and FFAs. All these products act as energy substrates and signaling molecules [75].

The β adrenergic/cAMP pathway impairs cell glucose uptake through insulin signaling by inhibiting mTOR (mammalian target of rapamycin) complexes (mTORC1 and mTORC2) and needs the presence of lipolysis products [76]. Thus, the increase of cAMP in fat cells due to β adrenergic stimulation surge (in severe burns) determines an inhibition of the PI3K/AKT/mTOR pathway, which is the insulin signaling pathway that mediates cell glucose uptake [76]. Furthermore, lipolysis products produce a complex dissociation of mTORC1 and mTORC2 which results in inhibition of this signaling pathway, hence blocking the insulin action of increasing glucose uptake into fat cells and other types of cells [76].

The elevated levels of circulating catecholamines are essential for acute stress response, but when these increased levels persist for months and even years, as it happens in severe burns, the effects upon the patient are deleterious [77].

#### 2.2.2. Cortisol

Cortisol (a glucocorticoid hormone produced by adrenal glands) is another well-known “stress hormone.” Its levels increase abruptly in severe burns and may persist for up to three years post-burn [12]. Cortisol has a low molecular mass and a lipophilic structure (it is derived from cholesterol), so it easily passes through the plasma cell membrane and gets into the cytosol, where the majority of glucocorticoid receptors (GRs) are, in an inactive form, bound by specific proteins [78]. After ligand binding, GRs lose the associated proteins and the activated cortisol–GR complexes enter the nucleus, where they bind to glucocorticoid-responsive elements (GREs) [79] (Figure 2). The result is the activation or inhibition of transcription of specific genes that codify the synthesis of regulatory proteins [80]. These are called genomic effects of cortisol and develop progressively [81].

There are also non-genomic effects of cortisol that develop rapidly (seconds, minutes). These are usually anti-inflammatory and immunosuppressive effects [80,82,83]. In stress situations, including severe burns, cortisol actions on glucose metabolism aim at furnishing glucose as an energy substrate for vital organs: the heart and the brain.

In hepatocytes, cortisol stimulates gluconeogenesis from glycerol and amino acids and also enhances glyceroneogenesis by increasing the expression of the gene encoding the PEPCK (phosphoenolpyruvate carboxykinase), a limiting factor of the rate of both of the aforementioned pathways [84,85].

In adipocytes, cortisol suppresses expression of the gene encoding the PEPCK, reducing glyceroneogenesis, which determines a decrease in fatty acids recycling and an increase in FFAs in circulating blood. Another effect of cortisol upon adipocytes is direct activation of lipolysis, which also contributes to an increase of FFAs and of glycerol, too, the latter being used for gluconeogenesis in hepatocytes [86].

In skeletal muscles, the main glucose consumer, cortisol inhibits glucose uptake and glycolysis and also obstructs glycogen synthesis through inhibition of insulin signaling and inhibition of activity of the glycogen synthase [87,88,89]. Furthermore, cortisol facilitates the glycogenolysis promoted by catecholamines [87]. The inhibition of glucose uptake is due to the fact that cortisol decreases the insulin-induced translocation of GLUT4 (glucose transporter type 4) to the cell membrane [89,90]. Moreover, by enhancing proteolysis in the muscle and glutamine synthesis, cortisol activity results in providing amino acids for gluconeogenesis [91].

The elevated levels of catecholamines, glucagon, and cortisol determine increased cycling of glucose and FFAs [92]. These processes are energy consumers and contribute to a greater energy expenditure, which is one of the characteristics of the hypermetabolic state of patients with severe burns.

### 2.3. How Glucagon and Insulin Modulate Glucose Metabolism in Burns

#### 2.3.1. Glucagon

Just like catecholamines, glucagon is elevated in situations of stress [93], including in severe burns. This pancreatic hormone produces hyperglycemia directly and exerts indirect actions on glucose metabolism through stimulation of lipolysis [94] and proteolysis [95]. At high concentrations, it increases both glucose uptake and lipolysis in human adipocytes [94,96,97]. In the liver, glucagon stimulates glycogenolysis and promotes gluconeogenesis [98]. Moreover, it is a ligand for class B GPCRs, which are mainly distributed in the liver and kidneys. These receptors are less expressed in the heart, pancreas, digestive tract, spleen, adipose tissue, cerebral cortex and are not expressed at all in muscles [99]. Glucagon receptor stimulation determines activation of adenylyl cyclase with consequent accumulation of cAMP and calcium in the cytosol. The increased cAMP activates PKA, leading to activation (through phosphorylation) of the GPK (glycogen phosphorylase kinase) which amplifies the breakdown of glycogen (glycogenolysis) in the liver [66,67] and increases the production of glucose-6-phosphate (G6P), further converted into glucose. Another effect of increased PKA activity (by glucagon) is the stimulation of phosphorylation of the CREB (cAMP response element-binding protein) with subsequent upregulation of the PEPCK transcription, thus stimulating an initial step of gluconeogenesis in the liver, namely, the conversion of oxaloacetate into phosphoenolpyruvate [100,101,102]. Hence, increased levels of glucagon in severe burns determine increased gluconeogenesis.

Glucagon decreases the consumption of glucose in the liver by inhibiting glycolysis through (1) inhibiting phosphofructokinase-1 (PFK-1) by reducing the levels of fructose-2,6-bisphosphate [103] and through (2) inhibition of the pyruvate kinase [104]. Glucagon’s actions concur towards increasing the blood levels of glucose as the prime source of energy in severe burns.

#### 2.3.2. Insulin

Insulin, a hormone produced by β-pancreatic cells, has anabolic effects upon glucose, lipid, protein, and energy metabolism [67].

##### Molecular Mechanisms of Insulin Action

The biological actions of insulin begin when it binds to its receptor, an integral glycoprotein composed of two subunits, α and β. The α subunit presents extracellular localization and represents the binding site for insulin, while β subunit is formed by a transmembrane domain and an intracellular tyrosine kinase domain activated by autophosphorylation. The two subunits are linked by disulfide bridges [105].

After the binding of insulin to its receptor with tyrosine kinase intrinsic activity, α-subunit suffers conformational changes and its catalytic function is activated, leading to autophosphorylation of Tyr residues from the β-subunit in the cytosolic region. Different adaptor proteins, such as IRS family members (IRS-1 and IRS-2), recognize auto-phosphorylated Tyr residues and activate two major signaling pathways, PI3K/AKT/mTOR involved in its metabolic actions (glucose transport, glycogen and protein synthesis, adipogenesis) and the mitogen-activated protein kinases/Ras pathway (MAPK/Ras) that regulates gene expression and insulin-associated mitogenic effects (gene expression, proliferation, differentiation, cell growth) [105,106,107].

Six IRS isoforms are known (IRS-1–6), where IRS-1 and IRS-2 mediate most of the metabolic effects of IR activation. IRS proteins present an NH_2_-terminal pleckstrin homology (PH) and PTB (phosphotyrosine-binding) domains and long COOH-terminal tails with tyrosine and serine/threonine (Ser/Thr) phosphorylation sites. After binding of the IRS PTB domain to IR pTyr972, IR phosphorylates multiple IRS Tyr residues, leading to downstream signaling effectors which propagate and amplify the insulin response [108].

IRS proteins have more than seventy COOH-terminal serine/threonine phosphorylation sites, so they affect IRS activity and protein stability [108]. IRS phosphorylation is considered to be the major mechanism by which several stimuli cause insulin resistance. Tyrosine-phosphorylated residues from IRS proteins recruit PI3K heterodimers which contain a regulatory p85 subunit and a catalytic p110 subunit, an essential node in insulin signaling [108]. In the liver, glucose is released through GLUT2 (glucose transporter type 2), while GLUT4 (glucose transporter type 4) mediates glucose uptake in muscles and the adipose tissue [109].

In the liver, AKT triggers insulin effects, such as glycogen synthesis and the suppression of gluconeogenesis [110]. Moreover, activated AKT can regulate transcription of the target genes from gluconeogenesis (PEPCK and G6Pase) via Foxo-1 [110]. Foxo-1 is a transcription factor that increases the expression of key gluconeogenesis enzymes, while its upregulation leads to the increased conversion of incoming substrates in the liver to glucose. Normally, in the liver, Foxo-1 is retained in the cytoplasm in an inactive form by the action of AKT after its phosphorylation [109].

AKT presents three isoforms (1, 2, 3), the AKT 2 isoform having an important role in insulin metabolic actions, especially in muscles and the adipose tissue, where its activation (by phosphorylation) leads to glucose uptake [105,109]. The IR is regulated by phosphotyrosine phosphatase (PTP) which dephosphorylates Tyr residues, reducing its activity. Moreover, PTP-1B is an essential component of insulin action-regulating mechanisms [105]. Another molecular mechanism involved in IR regulation is phosphorylation of Ser/Thr residues from the β subunit. Alterations at these levels have been detected to be associated with insulin resistance in both humans and rodents [105,109].

Studies regarding PTP-1B roles carried out on knockout mice revealed that this enzyme augments insulin sensitivity and enhances receptor Tyr phosphorylation and is impervious to the development of obesity and insulin resistance induced by high-fat diets [105,111,112,113,114].

PKC (protein kinase C) phosphorylates the β subunit of IRS in different intracellular regions. Several Ser/Thr kinases, such as PKA, c-Jun amino-terminal kinase (JNK), and p38-kDa mitogen-activated protein kinase phosphorylate the IRS and decrease its activity because they may affect receptor conformation or access to Tyr residues [105]. JNK, mTOR, ERK1/2, SIK-2, and different PKC isoforms phosphorylate more than 70 potential phosphorylation sites from 230 IRS-located Ser/Thr residues [105,115]. This is the key mechanism of insulin signaling inhibition that affects IRS Tyr phosphorylation and triggers decreased PI3K activity, promoting its degradation [105,116,117].

It is well-known that insulin levels rapidly increase in severe burns [118,119] in a process called post-burn hyperinsulinemia [12]. Despite the increased levels of insulin, burned patients have persistent hyperglycemia [12,113,120]. In trauma (such as severe burns), cells lose their sensitivity to the insulin’s action, a situation described as post-traumatic insulin resistance [121]. In these patients, hyperglycemia, which seems to accentuate insulin resistance, does not parallel the hyperinsulinemia, which persists much longer after the normalization of plasma glucose levels [12].

Hyperglycemia in the acute phase after the burn “satisfies” the increased energetic substrate demand for healing. Indeed, after an initially decreased glucose uptake in normal skin and soft tissue and in the burn area, there is a persistently increased glucose uptake in such tissues [122]. Adipose tissue [123], skeletal muscles [119], and the liver [92] manifest insulin resistance with insufficient cellular glucose uptake, which further accentuates and maintains the hyperglycemia [92]. Persistent hyperglycemia is linked to higher muscle protein catabolism, poor wound healing, greater skin graft loss, increased length of hospitalization, more frequent infections, and higher mortality [27,124,125,126]. It also enhances the release of inflammatory cytokines (TNFα, IL-1β, IL-6, etc.) by macrophages, monocytes, and adipocytes [127]. These cytokines, together with cortisol, catecholamines, FFAs, and hyperglycemia accentuate and maintain the insulin resistance.

In severe burns, cells suffer because of a major energy substrate deficit, which triggers (1) increased secretion of stress hormones (catecholamines, cortisol, glucagon) to mobilize glucose from glycogen and augment gluconeogenesis; (2) increased production of insulin. It appears that hormones like catecholamines and inflammatory cytokines such as TNFα and IL-1β attenuate or “blunt” pancreatic insulin secretion, so the β cells of the pancreas produce less insulin than needed due to the cellular energy depletion specific to severe burns [128]; this phenomenon might be explained by the “damage” to β cells produced by stress hormones and/or inflammatory cytokines [129] or by alteration of GLUT2 expression in β cells induced by hyperglycemia and increased FFA levels [129,130], which are common traits in the hypermetabolic state of severely burned patients. In human β-pancreatic cells, there are three glucose transporters, GLUT1, GLUT2 and GLUT3, [131,132], with glucose entering human β-pancreatic cells primarily via GLUT1 [132]. In rodents, it seems that GLUT2 is the most important glucose transporter concerning glucose uptake in β cells as the first step in glucose-stimulated insulin secretion [133]. The surface expression of GLUT2 depends on its interaction with a lectin that binds a specific N-glycan. Hyperglycemia and increased FFAs inhibit the activity of glycosyltransferase, which determines the lack of complex GLUT2 N-glycan formation. Consequently, GLUT2 are internalized, impairing the glucose-stimulated insulin secretion [134]. Therefore, post-burn, insulin secretion is augmented (causing hyperinsulinemia), but less than one would expect in such a hypermetabolic state [135].

The issue of insulin resistance has been largely debated and there are many factors to this process characterized by an inappropriate response of cells to insulin receptor binding. In severe burns, development of insulin resistance accentuates the hypercatabolic status of the patient.

##### Insulin Resistance—Signaling Pathway

From the molecular point of view, insulin resistance means disruption in the cascade insulin receptor–tyrosine kinase–GLUT4 translocation [136]. “Stress-induced insulin resistance may in part be due to phosphorylation-based negative-feedback, which may uncouple the insulin receptor or insulin receptor-associated proteins from its downstream signaling pathways” [125]. From the total insulin-stimulated glucose uptake, only 10% occurs in the adipose tissue and the liver, while the largest part occurs in skeletal muscles, the main glucose consumer [137].

Adipose tissue products, such as non-esterified fatty acids (NEFAs), glycerol, hormones (leptin and adiponectin), and proinflammatory cytokines, are involved in insulin resistance development [138]. In muscles, retinol binding protein 4 (RBP4) reduces the phosphatidylinositol-3-OH-kinase activity, and in the liver, it enhances the expression of the glucogenic enzyme PEPCK, leading to insulin resistance [138,139].

**Figure 3:**
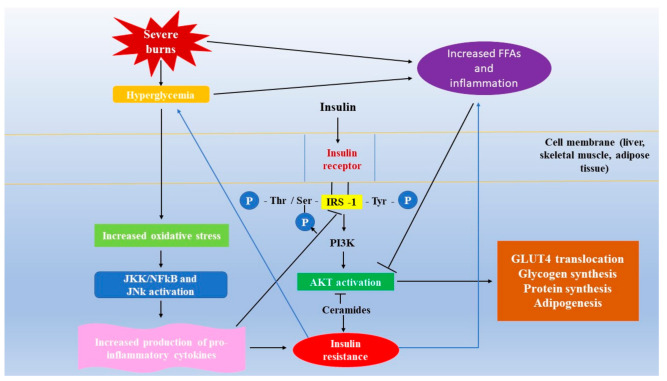
Insulin resistance in severe burns. Severe burns are characterized by hyperglycemia, which further promotes oxidative stress (OS) by increased production of reactive oxygen species (ROS), activating the JNK and IκB kinase-β (IKK-β)/(NF-κB) pathways, and increased production of proinflammatory cytokines. After the binding of insulin to its receptor, receptor autophosphorylation takes place, and members of the insulin receptor substrate (IRS) family are phosphorylated on Ser/Thr and Tyr residues. Phosphoinositide 3-kinase (PI3K) further activates protein kinase B or AKT, promoting GLUT translocation to the cell membrane, protein and glycogen synthesis, and adipogenesis. Severe burns are characterized by lipolysis, determining an increased production of free fatty saturated acids (FFAs) and ceramides, which have an inhibitory effect on the PI3K/AKT/mTOR signaling pathway, hence inducing insulin resistance. “
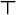
” shows an inhibitory effect.

Moreover, adipocyte-derived factors, such as the increased release of TNFα, IL-6, monocyte chemoattractant protein 1 (MCP-1), and additional products of macrophages, and other cells that populate the adipose tissue are involved in insulin resistance [138,140,141]. TNFα and IL-6, through classical receptor-mediated processes, stimulate both the JNK and the IκB kinase-β (IKK-β)/(NF-κB) pathways, leading to upregulation of inflammation mediators, and further to insulin resistance [138,140,141] (Figure 3). Key enzymes from glycolysis (pyruvate dehydrogenase, phosphofructokinase, and hexokinase) are inhibited by increased intracellular NEFA levels because these fatty acids may be in competition with glucose for substrate oxidation [138,142].

Fatty acid metabolism imbalance leads to increased intracellular levels of DAG, coenzyme A, fatty acyl coenzyme A, and ceramides, which further activate a serine/threonine kinase cascade and generate Ser/Thr phosphorylation of IRS-1 and IRS-2, resulting in a reduced ability of these molecules to activate PI3K [138,143]. DAG and ceramides activate inflammatory messengers such as PKCδ and induce impairment of the insulin signaling pathway by inhibition of IRS-1 Ser phosphorylation [138,144]. Increased levels of FFAs phosphorylate Ser residues from IRS proteins, decrease IRS Tyr phosphorylation, leading to impairment of downstream effectors [145]. Amino acids, mTOR, p70S6 kinase, hyperinsulinemia, JNK, stress, hyperlipidemia, inflammation, TNFα, obesity, mitochondrial dysfunction, hyperglycemia, and DAG cause IRS-1 Ser phosphorylation [106]. These molecular perturbations lead to the reduction of AKT phosphorylation and glucose transport into the cells [106,138,144].

Studies performed on insulin-resistant rodent models demonstrated Ser hyperphosphorylation of IRS-1 on Ser302, Ser307, Ser612, and Ser632 [106,146,147,148,149,150,151]. At the same time, in vitro studies are in concordance with in vivo studies and confirm that Ser phosphorylation conduces to the dissociation between the insulin receptor/IRS-1 and/or IRS-1/PI3K, preventing PI3K activation or increasing the degradation of IRS-1 [151,152,153,154]. Other factors such as endoplasmic reticulum stress, OS, aging, and hypoxia induce insulin resistance as well [155,156,157,158].

## 3. Current Clinical Strategies in Managing Metabolism Status/Glucose Metabolism after Severe Burns

### 3.1. Maintaining the Core Temperature of the Patient

The ambient temperature is important in the operating theater, during the surgical debridement, and in the intensive care unit where the patient is lodged. Hypothermia represents a stress and induces shivering in order to increase the body temperature. The immediate consequence is abrupt augmentation of the basal metabolic rate (BMR) [159]. The heat loss is increased during the change of dressings and during surgical excision. In order to prevent that, the ambient temperature must be higher for severely burned patients than for other types of patients [160].

On the other hand, excessive warmness in the intensive care room determines an increase of the core temperature of the patient, with subsequent increased perspiration and dehydration.

In order to prevent the increase of the BMR and, at the same time, to prevent the excessive water loss, it has been demonstrated to be optimal to maintain a core temperature of the patient above 37.5 °C [159] by ensuring an ambient temperature between 29–32 °C [160].

### 3.2. Sedation and Pain Control

Sedation and pain control are needed during surgery, garments change, on ward, and during physical therapy [161]. Uncontrolled pain of a burned patient represents an acute and extremely unpleasant sensation which also generates unwanted consequences such as persistent anxiety, posttraumatic stress, delayed wound healing, lack of compliance of the patient with the treatment and physical therapy, as well as increased rest energy expenditure [162].

For dressings change, ketamine is the preferred drug for intravenous analgesia and sedation [163]. The day-by-day control of severe pain is obtained with opioids associated with dexmedetomidine (a sedative drug) [161]. This association permits reduction of the dose of opioids and prevents the phenomenon of tolerance [164].

In order to reduce anxiety, it is recommended to administer benzodiazepine on a daily basis [165]. Meanwhile, for chronic pain treatment, synthetic opioids are favored, the best results so far being obtained with methadone [161]. Neuropathic pain is controlled with gabapentin [166].

### 3.3. Nutritional Support

There are two targets in the nutrition of severely burned patients, to provide enough nutrients in order to satisfy the increased caloric and protein requirements and to prevent the damage of the intestinal mucosa with subsequently augmented bacterial translocation from the gut to the blood. The caloric requirements in severe burns are dramatically increased [167] due to the hypermetabolic state [168]. The most accurate method to calculate the caloric need of a patient is indirect calorimetry, which is very difficult to use on a daily basis in burned patients [169]. This is why different formulas that approximate the caloric requirements were introduced: Curreri, Benedict, Toronto, Galveston, etc., none of them being perfect [168]. Prevention of the damage of the intestinal mucosa and bacterial translocation is achieved through early enteral feeding [170] usually done initially through jejunostomy and later through normal oral feeding.

In conclusion, the general principle of nutritional support in severe burns consists in rapid conversion (as soon as digestive tolerance develops) from parenteral nutrition to enteral nutrition [168] and low-fat (25%), high-carbohydrate (55%), and moderate-protein diets (20–25%) [171,172,173]. The nutritional formula used must include supplements with vitamins A, C, D, E, folic acid [174], and trace elements of zinc, copper, selenium, manganese, and iron [175,176].

### 3.4. Early Excision of Burn Wounds

The early and preferable total excision of burn wounds followed by coverage of the defects (grafts, skin substitutes, flaps, etc.) [177,178,179] is the mainstay in the treatment of burns [180].

Early excision and coverage of burn wounds reduce the systemic inflammatory response, mitigate the development of the hypermetabolic state, reduce water, ion, and protein loss, reduce the rate of septic complications and the hospitalization period, and decrease mortality by at least 20% [177,180].

Total excision of burn wounds is limited by two factors: blood loss and hypothermia [159]. Blood is lost from the excised areas and from the donor areas. In order to reduce massive blood loss, an alternative to total excision and skin grafting is represented by early staged excision and skin grafting or excision and covering with skin substitutes [178,179]. The strategy of excision and coverage should be adapted to each and every patient accordingly to the severity of the burn, associated morbidities, experience of the burn team, and experience of the intensive care team.

### 3.5. Physical Exercise

Early and continued physical exercise is mandatory in patients with severe burns [181]. Physical exercise improves the cardiorespiratory function [182], contributes to restoring of the lean body mass, and increases the function of skeletal muscles [183]. The physical exercise should start during hospitalization and continue after the patient is discharged for years [184].

The rehabilitative program is to be adapted to the specific needs of each burned patient and must be performed under qualified supervision.

### 3.6. Psychological Support

Burn trauma and the entire process of treatment have a deep impact on the patients’ mental health. There is a demonstrated causative connection between burn injuries and depression, anxiety, posttraumatic stress disorder, poor body image, sleep disorders, substance abuse, psychotic episodes, etc. [185].

This is why the victims of major burns and their families need qualified psychological support [185].

## 4. Pharmacologic Modulation of Glucose Metabolism in Burns

There is in fact a pharmacologic modulation of the hypermetabolic status of severely burned patients. Drugs represent a tool that improves the survival rates in severe burns, especially when used together with other therapeutical measures enumerated above.

Many drugs have been used to modulate glucose metabolism in burns, but only few proved to be efficient and are currently in use.

### 4.1. Insulin Therapy

The conventional (or submaximal) insulin therapy aims to decrease blood glucose levels, not at fasting levels (euglycemia), but around 130–150 mg/dL [186], in order to minimize the risk and frequency of hypoglycemic episodes [187]. It was proved that conventional insulin therapy decreases proteolysis and dramatically increases skeletal muscle protein synthesis (by about 400%) [188], preventing muscle mass loss in burns [187].

Intensive insulin therapy (IIT) aims to maintain blood glucose levels at fasting levels, but has an increased frequency of hypoglycemic events and was proven to increase mortality in non-burned ICU (intensive care unit) patients [189]. On the other hand, in severely burned pediatric patients, IIT lowers the mortality [190]. Beside the beneficial effects on the glycemia, insulin therapy increases the synthesis of fatty acids, decreases the production of some proinflammatory cytokines [124], reduces the hepatomegaly, decreases liver enzymes’ levels [124], and ameliorates the mitochondrial function [191].

Thus, insulin therapy remains one of the pillars in controlling glucose metabolism alterations in burns [190], but needs appropriate monitoring of glycemia [192], hypoglycemia being the main drawback in IIT in burns [188,192]. However, there are drugs that, when administered together with insulin therapy, permit the usage of reduced doses of insulin in burned patients. There are two favorable outcomes of such combinations of insulin with other medicines: reducing the incidence of hypoglycemia during insulin therapy and achieving euglycemia with lower insulin doses [193].

### 4.2. Fenofibrate

Fenofibrate is a lipolysis agonist that acts as a ligand for the peroxisome proliferator-activated receptor-α (PPARα) which, when activated, determines the induction of genes that encode enzymes of CYP4 (cytochrome P450 family) that are responsible for fatty acids oxidation and other enzymes responsible for the hydroxylation of saturated and unsaturated fatty acids [194]. The main effect is the reduction of serum triglycerides. Meanwhile, fenofibrate also reduces insulin resistance [194,195]. Therefore, the association of these two drugs permits the use of decreased insulin doses, thus diminishing the risk of hypoglycemic episodes [193].

### 4.3. Glucagon-Like Peptide-1 (GLP-1) and Analogs

Endogenous GLP-1 is a gut hormone (derived from preproglucagon) that tunes the secretion of insulin accordingly to the ingestion of carbohydrates [196]. It stimulates insulin secretion, inhibits glucagon secretion and gastric motility (consequently delaying gastric emptying), and decreases food intake, inflammation, and apoptosis [196].

It was proved that exenatide, a synthetic GLP-1 analog, decreases the need for doses of exogenous insulin to control glycemia in burned children [197], while also having the advantage of administration by subcutaneous injection once a week.

### 4.4. Metformin

Metformin is a biguanide hypoglycemic agent that limits the hepatic gluconeogenesis, increases insulin sensitivity and peripheric glucose uptake (this action is considered arguable by other authors), increases nonoxidative glucose metabolism and hepatic oxidation of fatty acids, and reduces lipogenesis [198]. However, it appears to have little or no effect on glucose levels in euglycemic patients. In addition, although metformin decreases the gluconeogenesis, it does not reduce the protein breakdown in skeletal muscles, but increases the rate of protein synthesis [199,200].

In burned patients, this drug is as effective as insulin in lowering the plasmatic glucose levels and rarely causes hypoglycemia [201]. Meanwhile, it also has the advantage of oral administration, being useful for long-term glucose control in severely burned patients [201]. Nevertheless, special attention is needed in burned patients with impaired renal function as metformin may produce/precipitate lactic acidosis because its clearance from the blood is partially related to the organic cation transporters’ (OCTs) activity: OCT1 is highly expressed in liver, OCT2—in kidneys, OCT3—in muscles and adipocytes. OCTs mediate the metformin’s concentration in tissues: in mitochondria, metformin inhibits oxidative phosphorylation (inhibits complex I of the electron transport chain) [198,202] and increases the glycolytic flux [203], leading to lactic acidosis [203,204].

### 4.5. Sitagliptin

Sitagliptin is a dipeptidyl peptidase IV (DPP-4) inhibitor. DPP-4 is a serine protease that inactivates GLP-1 [196]. Inactivating DPP-4 results in the elevation of plasma levels of active GLP-1, which leads to stimulation of insulin secretion and release after meals, improved glucose tolerance, and reduced glucagon levels [196,205].

In burned patients, coadministration of sitagliptin and insulin results in decreased exogenous insulin requirements by an average of 33.9% [206]. The consequence is a better control of hyperglycemia and fewer episodes of hypoglycemia related to insulin administration. It also has the advantage of oral administration and reduced adverse reactions. On the other hand, as it is excreted mainly through the kidneys, sitagliptin use requires caution in patients with renal insufficiency.

### 4.6. Recombinant Human Growth Hormone (rhGH)

Recombinant human growth hormone (rhGH) has important anabolic effects in skeletal muscles and in the skin [207]. In pediatric burned patients, it blunts the hypermetabolic response, improves the skeletal muscle protein kinetics, and increases the lean body mass [208]. Moreover, it seems to have antiapoptotic effects [209]. The major peripheral effector of rhGH is, just like for the natural growth hormone (GH), insulin-like growth factor 1 (IGF-1) [200]. The most important drawback of the administration of rhGH in burns is represented by hyperglycemia [210,211]. Indeed, in adult burned patients, rhGH determines hyperglycemia and hypermetabolism, and in non-burned critically ill adult patients, it increases morbidity and mortality by 40% [212].

### 4.7. Beta Blockers

Catecholamines are primary inducers of the hyperdynamic circulatory state and hypercatabolic state in severely burned patients [13]. Administration of beta blockers (the most studied being propranolol) in burns has proven beneficial effects: it decreases the cardiac workload and reduces tachycardia [213], decreases the hypercatabolic state [214] by decreasing the excessive thermogenesis and resting energy expenditure, reducing peripheral lipolysis [214], reducing the fatty infiltration of the liver [215], reducing insulin resistance and improving glucose metabolism [216], switching the skeletal muscle protein metabolism from catabolism towards anabolism with a secondary increase of the lean body mass [213], improving the mitochondrial function, and reducing the endoplasmic reticulum burn-induced stress [216].

There are studies reporting that non-selective β-blockers (propranolol, atenolol) may have adverse metabolic effects: blunting the perception of hypoglycemia in patients receiving insulin treatment (especially in type 1 diabetes mellitus, but not exclusively) [217,218], increasing plasma triglycerides [218], amplifying insulin resistance [218,219].

On the other hand, β_1_-selective antagonists (celiprolol, carteolol, nebivolol, carvedilol, bevantolol) do not blunt the perception of hypoglycemia [217,218] and improve the serum lipid profile of dyslipidemic patients [218,219,220].

## 5. Conclusions and Future Directions

The alterations of glucose metabolism in burns involving more than 30–40% of the TBSA together with other metabolic changes, cardiovascular alterations, and the systemic inflammatory response represent a reaction to this extremely severe trauma. This global reaction ensures the survival of the patient and might be considered, until a certain point, a “physiological response” to trauma. However, the persistence of hypermetabolism, of the inflammatory response, of hormonal alterations, and of the increased sympathetic activity determine severe consequences for the patient, giving rise to severe complications and finally death.

The characteristic persistent hyperglycemia triggers an increase in free fatty acids and induces insulin resistance, which in turn accentuates hyperglycemia and contributes to the augmentation of FFAs. The increased FFAs accentuate insulin resistance, too. Hence, a pathologic positive feedback is established.

Beside the surgical treatment and the parenteral (and enteral) nutrition, the pharmacological modulation using associations of insulin and fenofibrate plus β1-selective antagonists or insulin and sitagliptin plus β1-selective antagonists represents a very effective tool for decreasing the hyperglycemia of burned patients. The pharmacological modulation of glucose metabolism and of the systemic inflammatory response represents a very rewarding field of research in order to prevent physiological exhaustion of the patient and its unwanted consequences.

## Figures and Tables

**Table 1:** Specific alterations in the “ebb” phase and in the “flow” phase in severely burned patients.

	“Ebb Phase”	“Flow Phase”
Plasma volume	decreased	increased
Vascular resistance	increased	increased
Renal filtration	decreased	decreased
Cardiac output	decreased	increased
Tissue perfusion	decreased	decreased
Metabolism	hypometabolism	hypermetabolism
Mitochondrial dysfunction	initiated	accentuated
Endoplasmic reticulum stress	initiated	accentuated
Glycolysis	ecreased	increased
Proteolysis	decreased	increased
Lipolysis	decreased	increased
Thermogenesis	decreased	increased
Resting energy expenditure	decreased	increased
Insulin resistance	no	yes

## Data Availability

Not applicable.
